# Attitudes and behaviors of adolescents with asthma and their parents toward influenza and COVID-19 vaccination: barriers and facilitators of uptake

**DOI:** 10.1186/s12887-026-06575-2

**Published:** 2026-02-26

**Authors:** Leman Tuba Karakurt, Hayrunnisa Bekis Bozkurt, Gizem Uslu, Fatma Bal, Nurhan Kasap, Özlem Cavkaytar, Mustafa Arga

**Affiliations:** Department of Pediatric Allergy and Immunology, Faculty of Medicine, İstanbul Medeniyet University, İstanbul, Türkiye

**Keywords:** Asthma, Vaccine, Influenza, COVID-19, Children

## Abstract

**Background:**

Asthma is one of the most common chronic diseases of adolescence and significantly increases the risk of complications from respiratory viral infections such as influenza and COVID-19. Vaccination is the most effective preventive strategy, yet uptake among adolescents with asthma remains suboptimal. Parental beliefs and vaccine hesitancy are crucial determinants of immunization.

This study aimed to evaluate the attitudes and behaviors of asthmatic adolescents and their parents toward influenza and COVID-19 vaccination, with a focus on barriers and facilitators of uptake.

**Methods:**

This study was conducted as a descriptive, cross-sectional study at the Pediatric Allergy and Immunology outpatient clinic between May and September 2022. Children aged 12–18 years with a physician-confirmed asthma diagnosis and their parents were consecutively recruited during follow-up visits. A structured questionnaire addressing demographic characteristics, vaccination history, knowledge, attitudes, and concerns was administered face-to-face by physicians working in the Pediatric Allergy and Immunology Department. Data were analyzed using descriptive and comparative statistics, with *p*<0.05 considered significant.

**Results:**

A total of 212 adolescents with asthma (median age 14 years; 52.8% male) and their parents were included. Only 23.6% had ever received influenza vaccination and 6.6% were vaccinated regularly. Overall, 87.7% and 81.6% of adolescents expressed willingness to receive influenza and COVID-19 vaccines, respectively. Parental vaccination status was the strongest determinant of adolescent vaccination behaviors. Parental influenza vaccination independently predicted adolescent influenza vaccination (OR 7.26, 95% CI 3.29–16.0), and parental COVID-19 vaccination predicted adolescent willingness to receive the COVID-19 vaccine (OR 48.53, 95% CI 12.26–192.18).

**Conclusions:**

Despite high willingness, influenza vaccination rates among asthmatic adolescents remain low, contrasting with high COVID-19 uptake. Parental influence and safety concerns, particularly about long-term effects, were decisive factors. Targeted education, stronger physician recommendations, and clear communication on vaccine safety are essential to improve coverage in this vulnerable group.

## Background

Asthma is one of the most common chronic conditions in adolescence, affecting about 10% globally [1]. Respiratory infections such as influenza and COVID-19 can worsen asthma and increase hospitalization risk. Studies show that asthmatic children represent up to one-third of pediatric influenza hospitalizations, and guidelines highlight asthma as a risk factor for severe outcomes in both influenza and COVID-19 [2–4]. Viral infections in these patients frequently trigger exacerbations, underscoring the need for preventive strategies [3].

Vaccination remains one of the most effective protection for asthmatic adolescents. Annual influenza vaccines reduce infection rates and asthma-related hospitalizations, while COVID-19 vaccines lower the risk of severe disease [3, 4]. Health authorities strongly recommend that asthmatic youth remain up-to-date on these immunizations [5]. Evidence confirms both safety and efficacy in this population, reinforcing the importance of improving uptake [5].

Despite these benefits, vaccination rates among adolescents with chronic conditions remain below optimal [6, 7]. Studies from North America and Europe report vaccination rates ranging from 20% to 55% among asthmatic children and adolescents, despite clear clinical benefit [8, 9]. In Türkiye, influenza vaccination uptake remains particularly low. A national survey found that only 8–10% of the general population reported regular influenza vaccination, and rates were not substantially higher among patients with chronic respiratory disease [10]. Among parents of asthmatic children, one-quarter (25.4%) reported vaccinating their child during the previous influenza season, despite physician recommendations [11].

Vaccine hesitancy is often linked to fears of side effects, doubts about efficacy, and misinformation spread through peers and social media [6, 7, 12, 13]. Parental influence is especially decisive: adolescents’ acceptance strongly mirrors parental beliefs and healthcare provider recommendations [13, 14]. In fact, adolescents’ own views on vaccine safety and necessity tend to mirror the opinions expressed by their parents and healthcare providers. Conversely, if a parent is vaccine-hesitant – for example, doubting a vaccine’s efficacy or safety – their child is far less likely to receive that vaccine [15]. A more recent multicenter study indicated that parental willingness to vaccinate increased modestly after the COVID-19 pandemic, but misconceptions about vaccine necessity and mild disease perception remained major barriers [16].

While the global evidence highlights consistent parental influences and suboptimal coverage, little is known about vaccine attitudes and behavioral intentions among asthmatic adolescents and their families in middle-income contexts such as Türkiye.

This study aims to address this critical knowledge gap by examining the psychosocial and behavioral factors influencing influenza and COVID-19 vaccination attitudes, behaviors, and barriers among 12–18-year-old adolescents with asthma and their parents in Türkiye, with the goal of identifying strategies to improve immunization rates in this vulnerable group.

## Methods

### Study design and setting

This descriptive, cross-sectional study was conducted at the Pediatric Allergy and Immunology outpatient clinic between May and September 2022. The study population consisted of children aged 12–18 years who had a physician-confirmed diagnosis of asthma and their parents. Exclusion criteria included the presence of severe neurological impairment, inability to complete the questionnaire due to cognitive limitations, and refusal to provide informed consent. Participants were recruited consecutively during routine follow-up visits. After obtaining written informed consent from parents and assent from adolescents, both were invited to complete a structured questionnaire administered face-to-face by physicians working in the Pediatric Allergy and Immunology Department. The study protocol was approved by the Istanbul Medeniyet University Ethics Committee (No:2022/0218 Date:13.04.2022) and conducted in accordance with the Declaration of Helsinki.

### Definitions

Vaccine willingness was assessed using direct yes/no items asking whether the adolescent would receive the influenza and COVID-19 vaccines this season. Parental willingness was similarly evaluated. Vaccine hesitancy and concerns were measured through structured items addressing perceived adverse effects, allergic risk, trust in vaccine safety, and general attitudes, each captured using binary response options. Because responses were single-item constructs, internal consistency coefficients (e.g., Cronbach’s α) were not applicable. All items referred to current-season vaccination decisions. Denominators for proportions were defined as the total number of respondents who answered each specific item.

### Statistics

Data were recorded in a secure database and analyzed using IBM SPSS Statistics for Windows, Version 25.0 (IBM Corp., Armonk, NY, USA). The sample size was determined based on the primary outcome of influenza vaccination uptake rate in children with asthma. Based on previous research in a similar pediatric asthma population which utilized a group of 200 patients to identify parental predictors of immunization [17], we calculated that a minimum of 196 participants would be required to detect a 20% difference in vaccination rates between groups with 80% power and a significance level (alpha) of 0.05. To ensure statistical robustness and account for potential missing data, a total of 212 participants were enrolled. Continuous variables were summarized as mean ± standard deviation or median (interquartile range) according to distribution normality, while categorical variables were presented as frequencies and percentages. The Chi-square test or Fisher’s exact test was used for comparisons between categorical variables. Independent samples t-test or Mann–Whitney U test was applied for continuous variables as appropriate. Independent variables for the multivariable logistic regression model were selected based on both clinical relevance and statistical screening. Initially, all demographic and clinical variables were evaluated using univariate analyses. Those demonstrating a statistically significant association (*p* < 0.05) or recognized as major determinants in the literature were subsequently entered into the final multivariable model to calculate adjusted odds ratios (aOR) and identify independent predictors of vaccination behavior.

## Results

**Fig. 1:**
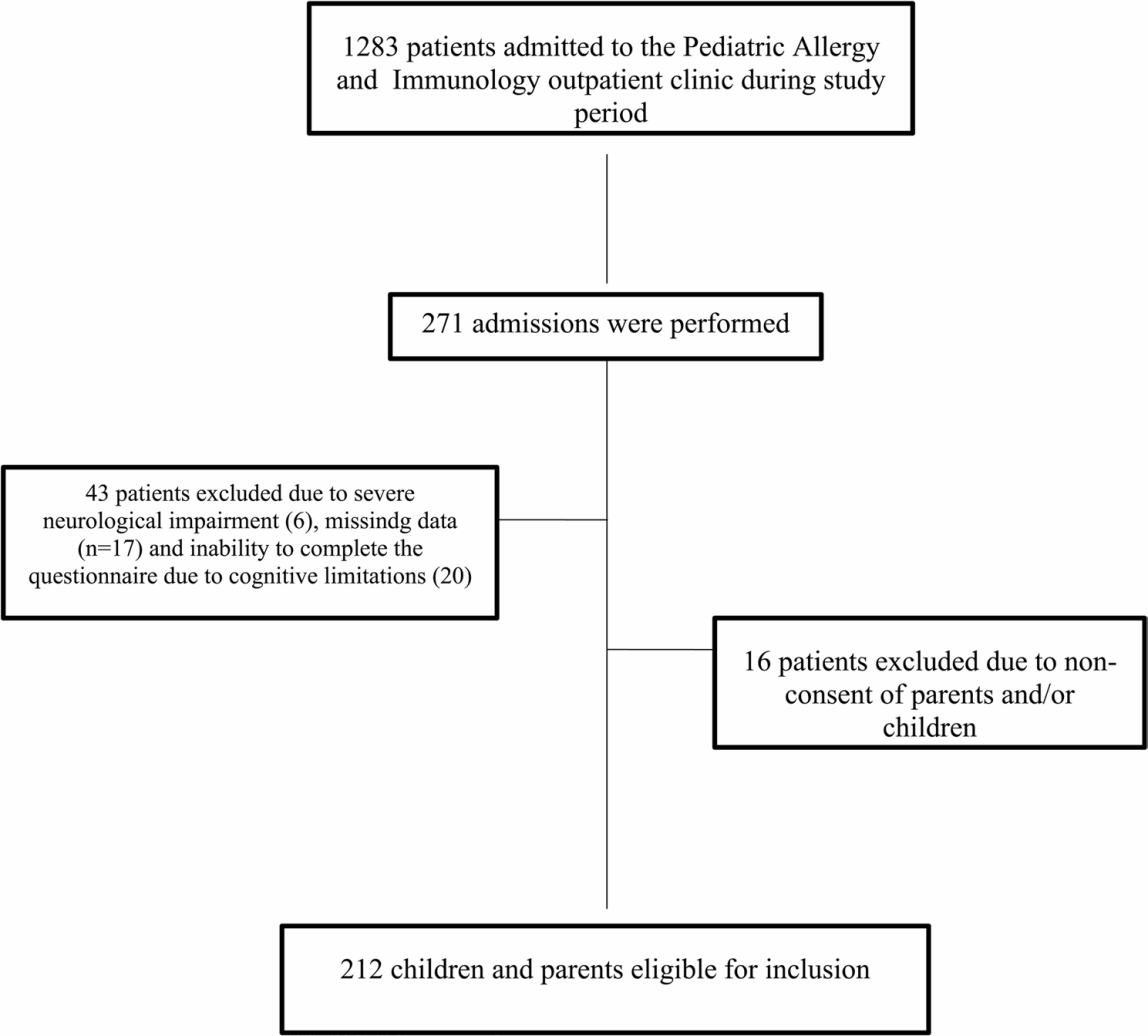
The flowchart of patients and parents enrolled in the study period

Overall 212 children with asthma and their parents were included in the study (Fig. 1). The median age of the children was 14 years (IQR 13–16), with 52.8% (*n* = 112) males (Table 1). The median age at asthma diagnosis was 6 years (IQR 3–16). Comorbid allergic diseases, such as allergic rhinitis, atopic dermatitis, or food allergy, were present in 63.7% of the patients (Table 1). Skin prick testing revealed atopy in 78.3% of the children. Regarding asthma control status, 80.2% were classified as controlled, 19.3% as partially controlled, and 0.5% as uncontrolled (Table 1). The median age of the parents was 42 years (IQR 30–65), and 73.6% (*n* = 156) were mothers. Educational levels were primary/secondary school (47.6%), high school (34.9%), and university (17.5%). Chronic disease and allergic disease were reported in 17.9% and 24.5% of parents, respectively.

**Table 1:** Demographic features, diagnosis and asthma control status of patients

Gender (*n*, %)	
Female	100 *(47.2)*
Male	112 *(52.8)*
Age (y) [median (IQR)]	14.0 (13.0–16.0)
Diagnosis age (y) [median (IQR)]	6.0 (3.0–16.0)
Comorbid allergic diseases (*n*, *%*)	
** +**	135 *(63.7)*
** -**	77 *(36.3)*
Skin prick test (*n*, *%*)	
Atopy **+**	166 *(78.3)*
Atopy **-**	46 *(21.7)*
Asthma control status (*n*, *%*)	
Controlled	170 *(80.2)*
Partially controlled	41 *(19.3)*
Uncontrolled	1 *(0.5)*

*IQR* interquantile range, *y* year

A total of 76.4% of children believed that asthma could increase the risk of severe influenza and COVID-19. Only 33% were aware that patients with asthma were prioritized for vaccination. COVID-19 infection in first-degree relatives was reported by 45.3% of participants. The infection rate was 13.7% among pediatric patients and 29.7% among parents.

Regarding vaccination history, 18.4% of parents and 23.6% of children had received influenza vaccination at least once, while regular influenza vaccination rates were 3.8% and 6.6%, respectively (Table 2). Also, 91% of parents were vaccinated against COVID-19, with 76.8% receiving mRNA (BNT162b2) and 23.1% receiving Sinovac. Post-vaccination side effects were reported by 55.2% of vaccinated parents, most commonly local reactions (83.0%), systemic symptoms (43.9%), and both local and systemic effects (16.9%) (Table 2).

**Table 2:** History of influenza and COVID-19 vaccination in children and parents, and post-vaccination side effects in parents

	Children [*n* (%)]	Parents [*n* (%)]
Influenza vaccination (at least once)	50 *(23.6)*	39 *(18.4)*
Regular influenza vaccination	14 *(6.6)*	8 *(3.8)*
COVID-19 vaccination	-	193 *(91.0)*
mRNA (BNT162b2)		148 *(76.7)*
Sinovac		45 *(23.3)*
Post–COVID-19 vaccination side effects	-	117 *(55.2)*
Local reactions		87 *(74.4)*
Systemic symptoms		21 *(17.9)*
Local + systemic		9 *(7.6)*

Overall, 87.7% (*n* = 186) and 81.6% (*n* = 173) of children expressed willingness to receive influenza and COVID-19 vaccine, respectively. The most common sources of information about vaccine eligibility were television (43.4%) and the national e-health portal (27.4%). Reported motivations for vaccination included being at higher risk due to asthma (50.3%), the desire to return to normal life (38.2%), physician recommendation (20.2%), and protecting vulnerable individuals (20.2%). Completion of routine childhood immunizations was reported in 99.1% of the children.

There was no significant association between asthma control and vaccination status. Influenza vaccination rates were similar between well-controlled and partly/uncontrolled asthma groups (24.7% vs. 19.0%, respectively) (*p* = 0.570) (Table 3). Likewise, willingness to receive the COVID-19 vaccine did not differ significantly by asthma control (82.4% vs. 85.7%, respectively) (*p* = 0.830). A significant relationship was observed between parental and child vaccination behaviors. Children of parents who had previously received the influenza vaccine were significantly more likely to be vaccinated against influenza compared to children of unvaccinated parents (56.4% vs. 16.2%, respectively; *p* < 0.001) (Table 3). Similarly, parental COVID-19 vaccination was significantly associated with adolescents’ willingness to receive the COVID-19 vaccine (89.5% vs. 14.3%, respectively) (*p* < 0.001) (Table 3). The presence of comorbid allergic or other chronic conditions was not associated with either influenza vaccination status (25.8% vs. 20.0%, respectively) (*p* = 0.430) or willingness to receive the COVID-19 vaccine (84.8% vs. 80.0%, respectively) (*p* = 0.590) (Table 3).

**Table 3:** The Associations Between Asthma Control, Parental Vaccination, Comorbidity and Child Vaccination Outcomes

	Child Vaccination Status					
	Influenza			COVID-19 willingness		
	+	-	*p*	+	-	*p*
Asthma control [*n*(%)]						
Well controlled (*n*=170)	42 *(24.7)*	128 *(75.3)*	0.570	140 *(82.4)*	30 *(17.6)*	0.830
Partly/uncontrolled (*n*=42)	8 *(19.0)*	34 *(81.0)*		36 *(85.7)*	6 *(14.3)*	
Parental influenza vaccination						
+ (*n*=39)	*22 *(56.4)*	17 *(43.6)*	<0.001	-	-	
- (*n*=173)	28 *(16.2)*	145 *(83.8)*		-	-	
Parental COVID-19 vaccination						
+ (*n*=191)	-	-		*171 *(89.5)*	20 *(10.5)*	<0.001
- (*n*=21)	-	-		3 *(14.3)*	18 *(85.7)*	
Concomitant chronic AD						
+ (*n*=132)	34 *(25.8)*	98 *(74.2)*	0.430	112 *(84.8)*	20 *(15.2)*	0.590
- (*n*=80)	16 *(20.0)*	64 *(80.0)*		64 *(80.0)*	16 *(20.0)*	

*AD* alergic disease

*Comparisons between categorical variables were performed using the Pearson chi-square test or Fisher’s exact test, as appropriate, based on expected cell frequencies

Comparisons demonstrated no significant difference in chronological age between adolescents who had received the influenza vaccine and those who had not (median 15.4 vs. 15.8 years, respectively) (*p* = 0.850) (Table 4). In contrast, diagnosis age was significantly higher among vaccinated children compared with unvaccinated children (median 7.0 vs. 5.0 years, respectively) (*p* = 0.046) (Table 4). Similarly, chronological age did not significantly differ between adolescents who were willing and unwilling to receive the COVID-19 vaccine (median 16.8 vs. 15.0 years, respectively) (*p* = 0.063). Diagnosis age was also comparable across these two groups, with no statistically significant difference observed (6.0 vs. 6.0 years, respectively) (*p* = 0.860) (Table 4).

**Table 4:** Comparison of chronological age and diagnosis age according to ınfluenza vaccination and COVID-19 vaccine willingness

	Age [median (IQR)]	*p*	Diagnosis Age [median (IQR)]	*p*
Influenza vaccination				
+	15.4 (12.0-16.5)	0.850	7.0 (4.0-10.0)	**0.046**
-	15.8 (12.8-17.3)		5.0 (3.0-8.0)	
COVID-19 vaccination				
+	16.8 (13.4-17.8)	0.063	6.0 (4.0-8.0)	0.860
-	15.0 (12.0-16.8)		6.0 (3.5-9.0)	

*IQR* interquantile range

Parental influenza vaccination was the only independent predictor of adolescents’ influenza vaccination (OR 7.26, 95% CI 3.29–16.0, *p* < 0.001) (Table 5). Similarly, parental COVID-19 vaccination was the sole significant determinant of adolescents’ willingness to receive the COVID-19 vaccine (OR 48.53, 95% CI 12.2–192.1, *p* < 0.001). Asthma control, gender, and comorbid allergic/chronic disease were not associated with either outcome in adjusted models.

**Table 5:** Univariate and multivariate logistic regression analyses of factors associated with influenza vaccination and COVID-19 vaccine willingness

	Influenza vaccination						COVID-19 vaccine willingness					
	Univariate			Multivariate			Univariate			Multivariate		
	OR	95% CI	*p*	OR	95% CI	*p*	OR	95% CI	*p*	OR	95% CI	*p*
Asthma control	1.39	0.60-3.25	*0.441*	1.17	0.47-2.91	*0.736*	0.80	0.31-2.09	*0.655*	0.92	0.30-2.76	*0.877*
Partly/uncontrolled(R)												
Well controlled												
Gender	0.78	0.41-1.46	*0.434*	0.82	0.41-1.64	*0.580*	1.21	0.59-2.51	*0.601*	1.54	0.63-3.75	0.340
Female (R)												
Male												
Parental influenza vaccination	6.70	3.16-14.20	**<0.001**	7.26	3.29-16.0	**<0.001**	2.74	0.79-9.47	*0.110*	2.23	0.56-8.85	*0.255*
- (R)												
+												
Parental COVID-19 vaccination	1.79	0.49-6.45	*0.377*	1.31	0.34-5.03	*0.696*	43.4	11.55-163.1	**<0.001**	48.53	12.2-192.1	**<0.001**
- (R)												
+												
Concomitant chronic AD	1.39	0.71-2.72	*0.340*	1.85	0.87-3.95	*0.110*	1.31	0.63-2.74	*0.469*	2.12	0.86-5.24	*0.104*
- (R)												
+												

*AD* alergic diseases, *CI* confidence interval, *OR* Odds-ratio, *R* reference

Concerns regarding influenza and COVID-19 vaccination varied between children and parents (Table 6). Among children willing to be vaccinated, 58.3% reported no hesitancy, whereas all children unwilling to be vaccinated expressed at least one concern. Similarly, only 11.9% of parents willing to vaccinate their children reported no hesitancy, compared to none in the unwilling group (Table 6). The most frequently reported concerns among children were short-term allergic reactions (23.1%) and other short-term side effects (18.5%), with higher rates observed among those unwilling to be vaccinated (28.2% and 33.3%, respectively) (Table 6).

**Table 6:** Concerns of children with asthma and their parents regarding COVID-19 vaccination

Concerns	Children willing to be vaccinated (*n*=173)	Children unwilling to be vaccinated (*n*=39)	Parents willing to vaccinate their child (*n*=176)	Parents unwilling to vaccinate their child (*n*=36)
No concerns	101 (58.3)	–	21 (11.9)	–
Risk of short-term allergic reactions	40 (23.1)	11 (28.2)	66 (37.5)	16 (44.4)
Risk of other short-term adverse effects	32 (18.5)	13 (33.3)	45 (25.5)	21 (58.3)
Lack of someone to answer vaccine-related questions	4 (2.3)	4 (10.2)	10 (5.1)	7 (19.4)
Allergic constitution / having asthma	36 (20.8)	8 (20.5)	–	–
Unknown long-term side effects	35 (20.2)	23 (58.9)	60 (34.1)	28 (77.7)
Uncertainty about duration of protection	5 (2.9)	10 (25.6)	21 (11.9)	7 (19.4)
Negative perceptions of the pharmaceutical industry	7 (4.0)	21 (53.8)	18 (10.2)	8 (22.2)
Rapid development of the vaccine	14 (8.1)	12 (30.7)	28 (15.9)	13 (36.1)
Negative comments in media (TV, social media)	14 (8.1)	12 (30.7)	70 (39.7)	13 (36.1)

Comparable findings were noted among parents: 37.5% of those willing to vaccinate their children were concerned about allergic reactions and 25.5% about other short-term side effects, increasing to 44.4% and 58.3% among unwilling parents.

Uncertainty regarding unknown long-term side effects emerged as the most prominent concern. While 20.2% of children willing to be vaccinated expressed this concern, the rate rose to 58.9% among those unwilling. Among parents, the corresponding figures were 34.1% and 77.7%, respectively (Table 6). Other reasons included having an allergic predisposition or asthma, rapid vaccine development, negative perceptions about the pharmaceutical industry, and negative information shared in the media. Concerns regarding the duration of vaccine-induced protection were also noted, ranging from 2.9 to 25.6% in children and 11.9–19.4% in parents (Table 6). Overall, all concerns were significantly more common among those unwilling to be vaccinated.

## Discussion

This study offers a concise yet meaningful contribution by examining both influenza and COVID-19 vaccine attitudes among asthmatic adolescents and their parents in a Turkish setting. Asthma substantially elevates the risk of complications from respiratory infections, highlighting vaccination as a critical preventive measure—a recommendation consistently endorsed by global guidelines [18, 19].

Despite these recommendations, uptake remains low globally; many countries consistently fail to achieve the WHO-targeted 75% coverage among high-risk groups, including individuals with chronic diseases and older adults, as documented in multiple studies across Europe and globally [20–22]. Among asthmatic children, influenza vaccination rates as low as 40% are reported [6]. Our study’s findings mirror this trend: only ~ 25% had ever received an influenza vaccine, with just 6.6% receiving it regularly. Nevertheless, nearly 88% expressed willingness to be vaccinated—indicating that uptake barriers likely stem from insufficient awareness or missed physician recommendations, rather than outright refusal [6, 18].

Our findings demonstrated a remarkably high willingness to receive the COVID-19 vaccine among asthmatic adolescents (81.6%), which surpasses many international reports. For example, in the United States, coverage among adolescents aged 12–17 years remained well below 70% in mid-2021, with only 42.4% having received at least one dose and 31.9% completing the series [23]. This likely reflects the acute public health urgency and extensive vaccination campaigns during the pandemic. Comparable reductions in hesitancy post-rollout have been observed in Türkiye and other settings, where initial hesitancy rates of ~ 30% dropped significantly as more safety data emerged [24].

Globally, safety concerns are the most common reason for vaccine refusal among both adolescents and parents [12]. In the current study, the primary driver of hesitancy was fear of long-term adverse effects (59% of children unwilling to be vaccinated and 78% of parents unwilling to vaccinate their child). In addition, concerns about allergic reactions were prevalent—understandable given the high rate of allergic comorbidities in asthma—and reflect patterns seen in similar populations [25–27]. Distrust in pharmaceutical companies and negative media portrayal also contributed to hesitancy, echoing findings from recent U.S. flu seasons where misinformation was linked to declining pediatric vaccination rates and increased severe outcomes [26, 28].

Adolescents’ vaccination behaviors closely tracked parental attitudes, consistent with evidence that parental health literacy and acceptance significantly influence youth immunization decisions [15, 29, 30]. Our participants primarily relied on trusted sources like physicians and e-health portals, underscoring the value of clinician engagement in vaccination promotion.

Previous studies have shown that influenza and COVID-19 vaccine uptake among adolescents with chronic respiratory diseases is strongly influenced by parental attitudes, perceived disease severity, and trust in vaccine safety, with parental vaccination emerging as one of the most consistent predictors of adolescent vaccine acceptance [31, 32]. Similarly, global reports indicate that asthma control alone does not reliably predict vaccination behavior, whereas family-level decision dynamics play a central role [33]. In our cohort, asthma control, gender, and comorbid allergic disease were not associated with either influenza vaccination or willingness to receive the COVID-19 vaccine, while parental vaccination status remained the primary determinant of both outcomes. These findings support the growing evidence that adolescent vaccination behaviors are shaped less by clinical severity and more by parental health behaviors and household-level attitudes toward vaccines.

This study’s strengths include its dual focus on influenza and COVID-19 within a high-risk, clinically defined asthmatic cohort; paired responses from adolescents and parents; and real-world vaccination data captured during clinic visits. These design features improve relevance and reduce social desirability bias compared to broader surveys.

However, this study has several limitations that need to be considered: (i) most importantly, vaccine hesitancy was not assessed using a standardized or validated instrument; although our questionnaire was developed based on established hesitation constructs and reviewed by experts in pediatric allergy, pulmonology, and public health, the use of a non-validated tool may limit the precision and cross-study comparability of our findings; (ii) it was conducted in a single tertiary center, which may limit the generalizability of the findings to broader or rural populations; (iii) cross-sectional design precludes any inference of causality between identified factors and vaccination behaviors; (iv) the survey did not include qualitative exploration, thus limiting deeper understanding of underlying beliefs and attitudes. Findings may not generalize to rural or more diverse populations, and causality cannot be assessed.

Despite these limitations, the data were collected using standardized clinical documentation, and internal consistency checks were applied to ensure data quality. Furthermore, while some determinants of vaccination behavior have been described in general pediatric populations, there is a lack of studies focusing specifically on adolescents with asthma in Türkiye. By providing disease-specific and region-specific insights, and by expanding the interpretation of comparative findings in the revised manuscript, our study offers meaningful contributions to the existing literature.

## Conclusions

This study reveals a critical gap in influenza vaccination among asthmatic adolescents, juxtaposed against high COVID-19 vaccine uptake. The main barriers to immunization are not opposition but rather concerns over safety, lack of awareness, and misinformation. Parental attitudes remain pivotal, with physician engagement and accurate information being key levers to improve uptake. These insights suggest that targeted educational interventions and stronger vaccine counseling in asthma care visits could substantially enhance protection against respiratory infections in this vulnerable population.

## Data Availability

The datasets generated and/or analyzed during the current study are available from the corresponding author on reasonable request.
